# Why do health-risk awareness and materialism drive consumers' acceptance intentions for smart green buildings?

**DOI:** 10.3389/fpsyg.2023.1238381

**Published:** 2024-01-26

**Authors:** Lydia Chu

**Affiliations:** Department of Architecture, National Taiwan University of Science and Technology, Taipei, Taiwan

**Keywords:** death anxiety, health-risk awareness, materialism, smart green building acceptance intentions, sustainable development

## Abstract

As the threat of global warming to human beings has gradually received attention, this article introduces the terror management theory to explain whether health risk awareness will affect their willingness to accept smart green buildings and that relationship is positively regulated by death anxiety. In addition, this article introduces the concept of materialism to propose that consumers will also accept smart green buildings due to the influence of materialism. This article interviewed two consumers and two professors in Taiwan, and verified the three hypotheses of this article through qualitative coding analysis. This article also used quantitative research methods to verify the theoretical model. This article not only introduces the cross-cutting contributions between terror management theory, materialism and smart green buildings, but also helps the construction industry develop strategies to attract consumers.

## 1 Introduction

Global warming has led to imbalances in ecosystems, species extinction, and threats to human life (Barbarossa et al., 2021), and the risk of disease occurrence and death have been spread through the media, which has also exacerbated people's stress, fear, and mental health anxiety (Yang et al., 2023). Relevant studies have pointed out that traditional non-intelligent green buildings account for 36% of global final energy consumption and 39% of total greenhouse gas emissions, making them the world's largest source of global warming (GlobalABC/IEA/UNEP, 2020). Therefore, smart green building is one of the concepts proposed to reduce the significant environmental, social, and economic impact of buildings (Machline et al., 2020).

In response to the above problems, this paper introduces terror management theory (Pyszczynski et al., 2015) to explain whether consumers' health risk awareness will affect their willingness to accept smart green buildings when they face the threat of climate change and death, and this relationship is positively moderated by death anxiety. Indeed, past studies have also pointed out that the research on the integration of terror management theory and climate change is not enough, so future research is needed to explore the mechanism of consumer behavior in different scenarios (Dalby, 2017). In addition, materialism is gradually spreading in Asian countries (Das and Jebarajakirthy, 2020), so this paper uses the self-completion theory (Hu et al., 2014) to explain why materialistic consumers have high willing nesses to accept smart green buildings. Based on symbolic self-completion theory (Hu et al., 2014), expensive luxury goods are important symbols of self-completion theory because they can highlight consumers' own success and material value. Compared with traditional non-smart buildings, smart green buildings are high-priced products, which have an effect similar to expensive luxury goods.

This paper interviews two consumers and two professors in Taiwan to confirm these three hypotheses and validates the research model of this paper through qualitative coding analysis. In addition, this paper also employs the quantitative research method to validate the theoretical model. This paper not only presents cross-cutting contributions between terror management, materialism, and smart green buildings but also assists the construction industry in developing strategies to attract consumers.

## 2 Literature reviewing

### 2.1 Health-risk awareness and acceptance intentions for smart green buildings

Terror management theory refers to the idea that individuals manage death anxiety and fear through two distinct systems of proximal and remote defenses (Pyszczynski et al., 2015). When an individual perceives that survival is threatened and that death may be imminent, proximal defenses are activated to suppress such thoughts to push death toward the more distant future by exhibiting behaviors that can reduce lethality (such as accepting green products to mitigation of future global warming). On the other hand, when the death perception is high enough to be on the verge of collapse, the remote defense will be activated. The individual will change his/her value to reduce the possibility of death. For example, a person changes his values in favor of green products and does not accept criticism and actions that violate these values (Pyszczynski et al., 2021).

Health risk perception refers to individuals' perception of the degree of pollution and harm caused by global warming to human health (Barnes and Dow, 2022). Based on the theory of terror management (Pyszczynski et al., 2015), global warming will cause individuals to perceive higher risk perception, which will lead to excessive fear perception, and may eventually produce proximal defense and remote defense mechanisms, thereby revising their values and demonstrating relevant green behaviors. Since traditional non-smart buildings are the main source of greenhouse gas emissions (GlobalABC/IEA/UNEP, 2020), according to terror management theory, individuals will have an acceptable behavior toward smart green buildings due to excessive health risk perception (proximal defense system). Moreover, these people also revise their values toward smart green buildings, because these actions can reduce the problem of global warming in the future (remote defense system), thereby reducing the threat of death. The first hypothesis proposed in this paper is as follows:

Hypothesis 1: Health-risk awareness positively affects acceptance intentions for smart green buildings.

### 2.2 Moderating effect of death anxiety

Death anxiety refers to the feeling of worry or fear that arises when an individual perceives a threat related to death (Askarizadeh et al., 2022). Because global warming will accelerate the possibility of human death, individuals will be afraid and have death anxiety (Vera et al., 2019). According to the terror management theory, when the awareness of health risks increases, the proximal defense will be activated. Individuals will have behaviors related to environmental protection (such as the acceptance of smart green buildings), and death anxiety may increase the degree of individual fear, thereby strengthening this mechanism. Similarly, when health risk awareness is raised to the brink of collapse, the individual's remote defense mechanism will be activated, and the individual will start to revise their original values and turn to green environmental protection, which is also strengthened by death anxiety. The second hypothesis proposed in this paper is as follows:

Hypothesis 2: Death anxiety positively moderates the relationship between health-risk awareness and acceptance intentions for smart green buildings.

### 2.3 Materialism and acceptance intentions for smart green buildings

Materialism refers to the degree to which an individual demonstrates success and happiness in life through material possession or wealth (Williams, 2020). The symbolic self-completion theory (Hu et al., 2014) can be used to explain the behavioral patterns of material consumers. Because high-priced products are an important symbol of consumers' self-completion success and life happiness, materialistic consumers are more willing to accept high-priced products. Past research has also found that materialistic consumers are more willing to accept high-priced products to flaunt their success and life happiness (Siahtiri and Lee, 2019). Therefore, consumers with a high degree of materialism should be more willing to accept smart green buildings than ordinary consumers, because the price of smart green buildings is higher than that of traditional buildings. The third hypothesis proposed in this paper is as follows:

Hypothesis 3: Materialism affects consumer acceptance intention for smart green buildings.

## 3 Methodology

### 3.1 Qualitative methodology

**Figure 1:**
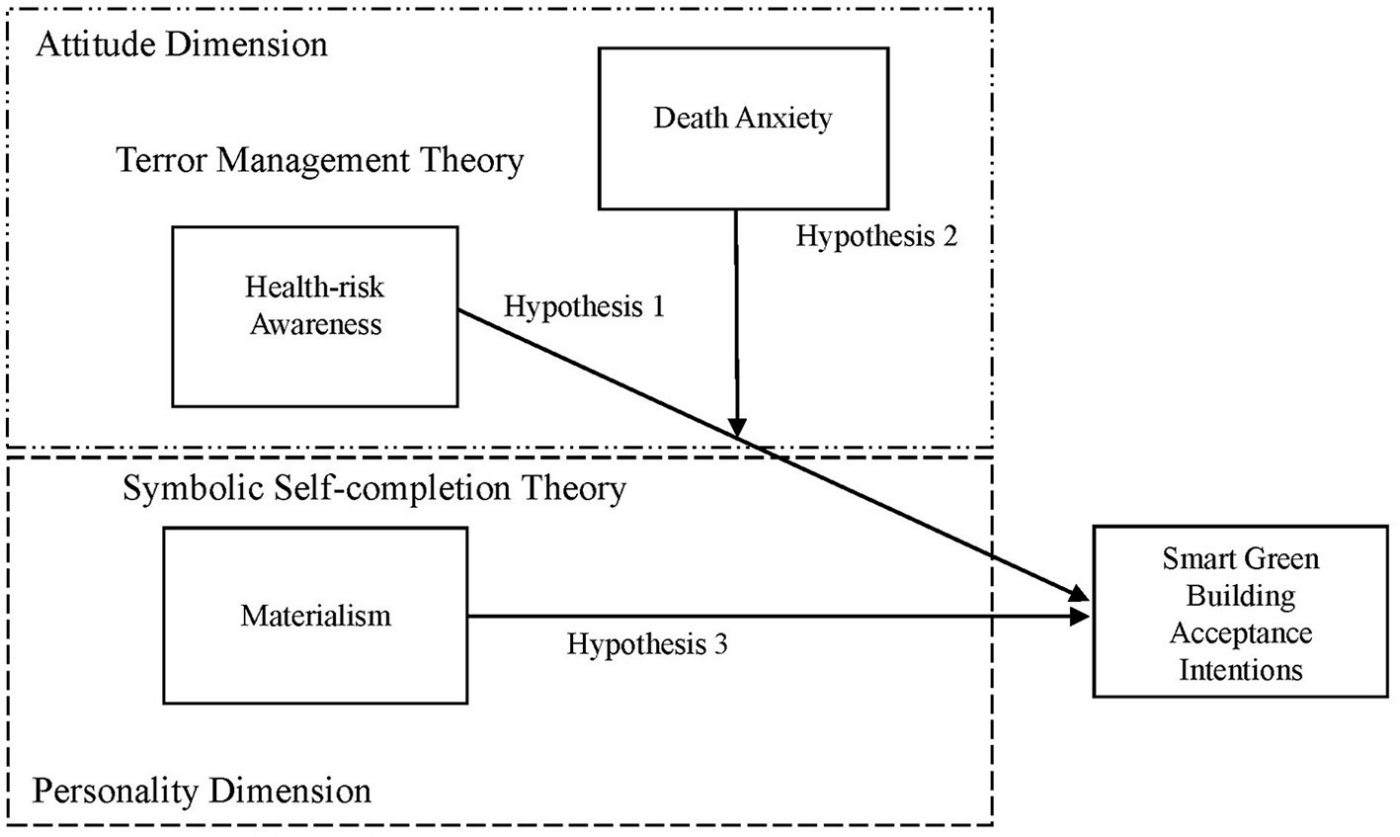
Theoretical model of this article.

Figure 1 depicts the research framework of this paper, which describes how attitude dimensions (health risk awareness and death anxiety based on terror management theory) and personality dimensions (materialism based on symbolic self-fulfillment theory) affect acceptance intentions for smart green buildings. To test the research framework in this paper, two professors and two consumers were interviewed to obtain a transcript for qualitative coding analysis. Cohen's kappa coefficient of qualitative coding is 0.81, meeting the reliability requirement (Trochim and Donnelly, 2001). Qualitative coding analysis confirms all hypotheses of this paper.

### 3.2 Quantitative methodology

#### 3.2.1 Measurements

This paper modified the scale from Richins's (2004) to measure materialism, and an example item is “It is …. money can buy happiness.” Acceptance intentions for smart green buildings were measured by Han and Hyun's (2017) scale, and an example item is “I would accept green smart buildings.” Health-risk awareness was measured by Wang et al. (2020) scale, and an example item is “Climate change, global warming … will threaten personal health.” Finally, death anxiety was developed by Rahimah et al.'s (2020) scale, and an example item is “When I think about how short life is, I get disturbed.”

#### 3.2.2 Sample collection

This paper recruited consumers who have purchased luxury goods in the past 6 months because this research wanted to measure the different levels of the materialism of these consumers and their behaviors, which is also adopted by previous empirical studies (He et al., 2016). This research obtained 304 consumers who are willing to participate in this survey.

#### 3.2.3 Validity and reliability

To validate the validity and reliability, this research adopted the analysis method of confirmatory factor to obtain Cronbach's α, composition reliability, and average variance extracted (see Table 1). The three indices are all greater than critical values (Fornell and Larcker, 1981). In addition, the model fit is also shown in Table 1.

**Table 1:** Validity and reliability.

**Variable**	**Cronbach's α**	**Average variance extracted**	**Composition reliability**
Materialistic value	0.55	0.69	0.96
Acceptance intention for smart green buildings	0.85	0.68	0.89
Health-risk awareness	0.84	0.79	0.86
Death anxiety	0.89	0.75	0.92

GFI = 0.90; CFI = 0.91; NFI = 0.90; RMSEA = 0.049; RMR = 0.048.

#### 3.2.4 Analysis results

Materialistic value positively affects acceptance intention for smart green buildings (coefficient = 0.55, *P* < 0.01), and health-risk awareness positively affects acceptance intention for smart green buildings (coefficient = 0.41, *P* < 0.01), which validates hypothesis 1 and 2. That said, consumers who have more materialistic value and health-risk awareness will demonstrate more acceptance intention for green smart buildings.

Next, death anxiety positively moderates the relationship between health-risk awareness and acceptance intention for smart green buildings (coefficient = 0.1, *P* < 0.01). That said, consumers who perceive more death anxiety will demonstrate acceptance intention for smart green buildings caused by health-risk awareness.

## 4 Discussion

### 4.1 Contribution

This paper uses terror management theory combined with symbolic self-completion theory to explain the willingness to accept smart green buildings. However, previous studies have paid little attention to how to increase consumers' willingness to accept smart green buildings, especially when traditional non-intelligent buildings are the main source of greenhouse gas emissions. Therefore, it is an important topic to explore the driving factors of the willingness to accept smart green buildings.

Next, the findings of this paper support the proposed hypothesis and fill in the gaps of previous studies. Past research has noted that death anxiety reinforces environmental behavior and its predecessors (Guerriero and Swim, 2022), but few studies have explored this gap more broadly. This article addresses this gap by considering death anxiety as a moderator between health risk perception and environmental behavior. Finally, the article fills in specific policy references on how the government can strengthen people's environmental behavior. Since government agencies are the main promoters of environmental policies, they can increase public awareness of the greenhouse effect and risk perception through the news media, thereby influencing public environmental behavior (such as acceptance of smart green buildings).

Finally, the paper also makes some contributions to the formulation of marketing strategies for the construction industry. First of all, because the acceptance of smart green buildings by material consumers is higher than that of ordinary consumers, material consumers are the main market for smart green buildings. Therefore, construction companies should devote marketing resources to materialistic consumers. For example, construction companies can combine well-known corporate brands in smart green building advertisements to increase the high-priced impression of buildings or combine celebrity endorsements to meet the needs of materialists.

### 4.2 Research limitations and future research

This paper used a four-person sample of qualitative interviews to initially test the three hypotheses, further research should use more sample of qualitative interviews to validate the hypotheses. Second, future research should use samples from different countries to verify the theoretical model of this article, because samples from different countries may have different effects on the variables in this study. For example, Asians and Europeans may have different perceptions of materialism and risk awareness, which may lead to different green consumption behaviors. Finally, the results of the analysis do not mean that the three hypotheses have been confirmed, so more longitudinal data should be examined to validate the causal relationship of theoretical model in this article.

## Data availability statement

The original contributions presented in the study are included in the article/Supplementary material, further inquiries can be directed to the corresponding author.

## Ethics statement

Ethical review and approval was not required for the study on human participants in accordance with the local legislation and institutional requirements. Written informed consent from the patients/participants or patients/participants' legal guardian/next of kin was not required to participate in this study in accordance with the national legislation and the institutional requirements.

## Author contributions

The author confirms being the sole contributor of this work and has approved it for publication.
